# What role does temporal synchrony play in mid-level audiovisual crossmodal correspondences?

**DOI:** 10.3758/s13423-026-02877-9

**Published:** 2026-03-17

**Authors:** Charles Spence, Nicola Di Stefano

**Affiliations:** 1 Crossmodal Research Laboratory, Department of Experimental Psychology, Oxford University, Oxford, OX1 3PS UK; 2 Institute of Cognitive Sciences and Technologies, National Research Council, Rome, Italy

**Keywords:** Synchrony, Gestalt perceptual grouping, Dynamic crossmodal interactions, Crossmodal correspondences

## Abstract

Temporal synchrony is widely recognized as one of the key factors facilitating the emergence of crossmodal correspondences and affecting their crossmodal effects. However, several issues regarding the definition of temporal synchrony and the mechanisms underlying its crossmodal effects remain open, depending on the specific experimental/perceptual context/stimuli used, as well as the influence of crossmodal congruency and structural (including isomorphic) crossmodal correspondences. In this review, we take a closer look at the literature that has been published in this area over recent decades in order to critically evaluate what is currently known concerning the crossmodal effects that are mediated by temporal synchrony. We focus especially on mid-level audiovisual crossmodal correspondences, defined as those that involve multi-element, or dynamic, auditory and visual stimuli. We examine the different experimental methodologies used and their limitations as well as the theoretical frameworks that have been proposed to account for the viewer’s impression of (and the meaning/affect that is associated with) such experimental audiovisual displays, including those that are based on the ‘Congruency-Associationist Model’, Gestalt perceptual grouping, as well as the phenomenon of multisensory emergence. Finally, we outline several directions for future research on temporal synchrony in the context of audiovisual crossmodal correspondences.

## Introduction

In recent years, there has been an explosive growth of interest in the topic of crossmodal correspondences (e.g., for reviews, see Motoki, Velasco, & Marks, 2023; Spence, 2011, 2018a). Crossmodal correspondences are the sometimes surprising crossmodal associations between features, attributes, or dimensions of experience, either directly perceived or else merely imagined, in different sensory modalities. For instance, people often intuitively choose to associate high-pitched sounds with bright, small, visual objects that are positioned high in space, while associating low-pitched sounds with darker, larger, visual objects that happen to be positioned lower in space (Evans & Treisman, 2010; Parise & Spence, 2012). Similarly, certain tastes, such as sweetness, are frequently matched with round shapes or soft textures, whereas bitterness tends to be linked with angular forms or low-pitched sounds and rough textures instead (for reviews, see Deroy, Crisinel, & Spence, 2013; Spence, 2023). These intuitive pairings are not only robust across individuals but have also been shown to influence perception, preference, and behaviour across a range of experimental and real-world contexts.

Recently, Spence and Di Stefano (2025a) introduced an important distinction between simple, mid-level, and complex crossmodal correspondences. According to this classificatory framework, simple crossmodal correspondences involve individual sensory attributes or dimensions, such as pitch, hue, or intensity (for reviews, see Marks, 2004; Spence, 2011); mid-level crossmodal correspondences involve combinations of (possibly dynamic) unisensory stimuli, such as short sequences of sounds (or video animations); and complex crossmodal correspondences involve semantically rich and/or emotionally meaningful complex stimuli, such as paintings, music, film clips, and other works of art (for reviews, see Spence, 2020; Spence & Di Stefano, 2025b). While the majority of research that has been published to date in this area has studied crossmodal correspondences occurring between stimuli at the same level of complexity, such as pitch-hue correspondences (though see Spence & Di Stefano, 2024), or crossmodal associations between music and paintings/drawings (e.g., Di Stefano et al., 2025; Di Stefano et al., 2024; for review, see Spence, 2020), a few researchers have also studied cross-level crossmodal correspondences; for example, consider here only the crossmodal correspondences that have been documented between classical music selections and colour patches by Palmer, Schloss, Xu, and Prado-León (2013; see also Hauck, von Castell, & Hecht, 2022; McDonald et al., 2022).

Empirical findings and theoretical reflections highlight the key role of temporal synchrony in binding the unisensory contents that are actually associated crossmodally (for reviews, see Vatakis & Spence, 2010; Vroomen & Keetels, 2010). In its narrowest sense, the term refers to the precise co-occurrence of sensory events, such as an auditory click and a flash of light, which is believed to promote multisensory integration. This millisecond-level simultaneity underpins many controlled experiments on audiovisual integration and appears to benefit from transient stimulus onsets (e.g., Andersen & Mamassian, 2008; Cook, Van Valkenburg, & Badcock, 2011; Raij et al., 2010; Van der Burg et al., 2010). However, it should be noted that such a definition is most readily applicable to those contexts involving discrete or intermittent events, where temporal alignment can be clearly marked and easily measured, such as in laboratory experiments using bouncing balls or flashing lights.

In everyday contexts, however, the perception of synchrony can be more flexible (or ambiguous): that is, visual and auditory stimuli can appear synchronized when they share a rhythmic pattern or common temporal structure, even if their individual elements do not exactly align (Di Stefano & Spence, 2025). For instance, dancers may align with the beat of a soundtrack only intermittently, and yet still appear ‘in sync’ due to the presence of periodic or expressive correspondences (Heins et al., 2021; Keller & Repp, 2008; Wakiyama, Tsubaki, Kuno-Mizumura, & Sakaguchi, 2025). Complicating matters further, synchrony can be attributed to or recognized in expressive or structural patterns in the stimuli across modalities. For instance, auditory and visual events might match in terms of their dynamic changes in tension, trajectory, or salience over time (Munárriz Ortiz, 2017). These occurrences of higher-order synchrony that involve looser, but nevertheless still perceptually compelling, mappings raise questions regarding the very notion of temporal synchrony and the extent to which precision in time is required to generate the desired crossmodal effect. Indeed, separately, other researchers have demonstrated how it may be the correlation between unimodal temporal stimulus patterns, rather than the exact temporal synchrony of the elements in each stream, that is key to multisensory integration (see Parise, Harrar, Ernst, & Spence, 2013; Parise, Spence, & Ernst, 2012).

In this review, we focus on the role of temporal synchrony in facilitating crossmodal associations and in mediating their crossmodal effects. We start by reviewing the key literature that has been published on mid-level crossmodal correspondences, with a particular focus on the temporal dimension of stimuli matching (Section "Critical review of research involving mid-level crossmodal correspondences"). Thereafter, we examine crossmodal grouping at a perceptual level, with the aim of identifying a number of organizing principles that recur across the empirical literature and constrain how audiovisual integration can occur (Section "Crossmodal grouping and perceptual organization"). In the "Discussion" section, we discuss the key methodological issues of the reviewed literature as well as their implications for theoretical reflections before outlining directions for future research in the area of audiovisual correspondences and temporal synchrony. Overall, this review suggests that a more nuanced account of temporal relationships in audiovisual perception is required, one that is capable of accommodating both low-level synchrony and higher-order structural and expressive relations underlying reported crossmodal effects.

## Critical review of research involving mid-level crossmodal correspondences

The majority of the studies targeting mid-level crossmodal correspondences that have been published to date involve studying the effect, if any, of adding different kinds of auditory and musical stimuli to visual stimuli. Several, at times overlapping, experimental literatures can be distinguished here: A number of researchers have, for instance, chosen to investigate the impact of (primarily classical) music on people’s ratings of static visual stimuli (often consisting of pictures of paintings; for a review, see Spence, 2020). Meanwhile, a separate empirical literature has emerged documenting the impact of music on people’s perception of dynamic visual film clips (for a review, see Spence & Di Stefano, 2025c). Meanwhile, a third body of research has investigated more synaesthesia-like connections between auditory and visual stimuli, often in the context of what might be called multimedia art (e.g., Daniels, Naumann, & Thoben, 2010; Deutsch, 2012; Kargon, 2011; Toccafondi, 2025; Zika, 2013).^1^

**Fig. 1:**
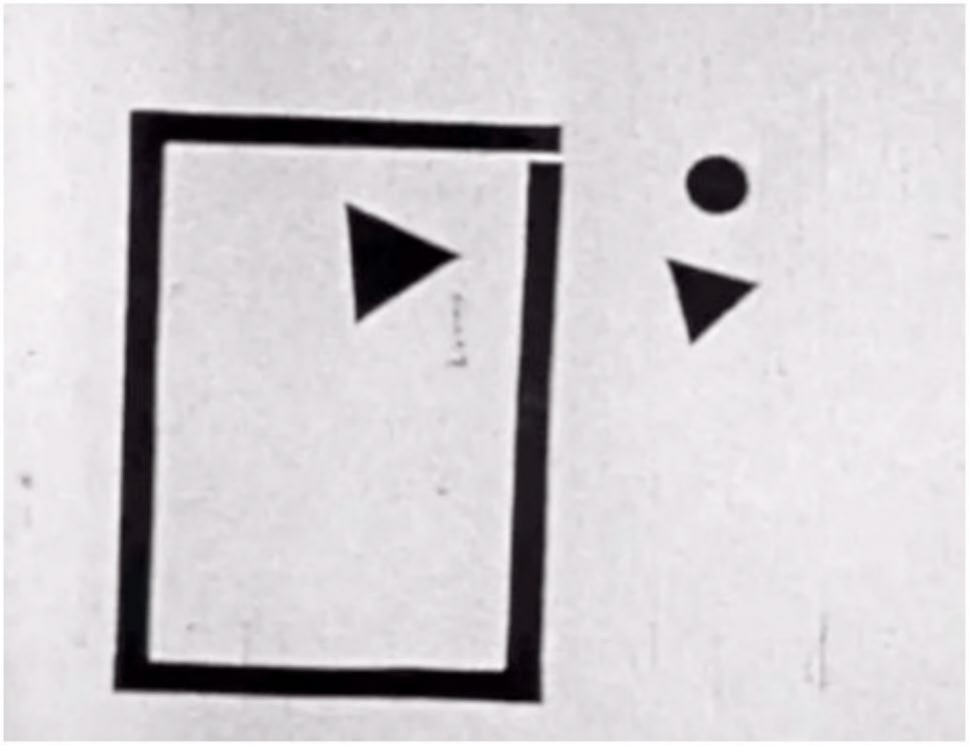
Screenshot of the film figures (Heider & Simmel, 1944)

In one early study, Marshall and Cohen (1988) presented their participants with a 2-min abstract animation showing a large and a small triangle and a small circle in motion (see Fig. 1). Typically, when people view this short animation, they tend to interpret the large triangle as a bully who is victimizing the two smaller geometric figures (the small triangle and small circle). Marshall and Cohen studied the effect of two contrasting pieces of background music played while participants viewed this film clip on the latter’s attitudes toward the figures shown in the animation. Baseline information about the meaning of the music and visual stimuli was first obtained on 12 bipolar adjective rating scales (e.g., powerful/powerless). For the visual stimuli, the participants had to judge each of the three stimuli individually, and then provide an overall evaluation of the film as a whole. Next, a new group of participants was simultaneously presented with one combination of music plus film, and once again had to judge the film ‘characters’ and the film overall on the same 12 bipolar adjective ratings. In total, there were five groups of five participants in the preliminary study, with each group being presented with the same stimulus twice. The first time round the participants had to describe what happened in the film and supply adjectives to describe the three shapes or the music. The participants were also asked whether the music reminded them of anything. The second time through, the participants were instructed to assess each figure and/or the music on 13 rating scales.

In Marshall and Cohen’s (1988) main study, two originally composed contrasting but structurally similar musical accompaniments were created. They incorporated three main musical ‘themes’ used at specific points in the visual animation. One theme accompanied the introduction of the large triangle, the second theme coincided with the large triangle contacting the small triangle, and the third auditory theme was introduced when the small shapes are ‘chased’ around the rectangular enclosure by the large triangle. One version of the music was in major mode, the other in minor mode. The former was classified as ‘weak’, the other as ‘strong’. A no-music baseline condition was also presented. In the main study, Evaluation, Potency, and Activity, were once again assessed using the Semantic Differential technique (with four bipolar adjective scales for each dimension). Intriguingly, judgments of the individual shapes differed for a given musical accompaniment. So, for example, the activity ratings for the three geometric characters were shown to differ significantly under the two music backgrounds, although the overall activity rating of the two pieces of music did not differ significantly.

Marshall and Cohen (1988) attempted to explain these results by suggesting that an interaction had taken place between the temporal structure of the film and music that influenced the participants’ visual attention, such that its focus differed under the two musical conditions (that said, no objective measure of participants’ fixation was provided to back up such post hoc claims, though note that covert shifts of attention would not be observable using such techniques either) (see Table 1 for a summary of results). The sample size (namely five participants per condition) in Marshall and Cohen’s preliminary study was likely statistically underpowered by today’s standards; such a limitation is all the more important given the recent replication crisis in (psychological) science (see Bohannon, 2015), and the absence of any attempts at replication in this area that we are aware of. Nevertheless, Marshall and Cohen suggested that congruence in terms of the three principal dimensions of the semantic differential (Evaluation (good/bad), Potency (strong/weak), and Activity (active/passive)) likely influenced the meaning of the stimuli in this study and were computed simultaneously in each modality. In particular, different ratings on the calm-agitation dimension of the film figure as a function of the sound that was paired with the animation suggest that sound influenced the meaning of the film characters for the participants.

**Table 1:** Chronological summary of studies involving audivisual mid-level crossmodal correspondences (typically involving visual abstract animimations paired with either dynamic configurations of sounds or pre-composed music clips)

Study	N (participants)	Audiovisual Stimuli	Results
Marshall & Cohen (1988)	25 psychology students; 5 per condition; Stimuli presented twice	2-min Heider & Simmel (1944) abstract animation; Prelminary study: *Adagio* (weak) & *Allegro Marcato* (strong) section from Prokofiev's Symphony No.5. Main study: Major (Weak) or Minor (Strong) compositions.	Activity ratings for three characters in animation changed by the presence of music. E.g., activity rating of small triangle increases with strong music as compared to weak music or no music.
Cohen (1993)	E1: 12 participants	Video of bouncing ball varying in height and speed of bounce; Repeating melodic tone varying in pitch height and tempo.	Pitch height and tempo of music influenced participants' ratings of the happiness/sadness of the visual animations.
Sirius & Clarke (1994)	10 students x 4 films, 8 rate visual only, & 9 rate music only	Abstract video animation; 4 pieces of music composed in Disco, Spanish, Thriller, & Epic music (i.e., 'Spaghetti Western') styles.	Music exerted a consistent, additive effect on the evaluation of visual stimuli, buit no interaction effects observed.
Iwamiya (1994)	E1: 9 students	40 video disc excerpts, 20 matching & 20 mismatching to some degree (either desynchronized or mismatching).	Audition affected vision for higher-level factors ('cleanliness' & 'uniqueness') for matching stimuli; Brightness and evaluation also affected.
Lipscomb & Kim (2004)	28 participants	Rate degree of 'match' for 48 audivisual composites, varying in e.g., tempo, pitch, duration, & size.	Pitch associaed with vertical location, loudness with size, & timbre with shape.
Kim & Iwamiya (2008)	E1: 13 students	Nonsense letter strings projected on screen ('Telops'); Range of sound effects from broadcast programs in Japan (e.g., 'noise burst with gradual attack and delay; two consecutive Japanese drum sounds).	Formal audiovisual congruency led to higher ratings of audivisual stimuli: In particular, synchronous audiovisual onset and/or matching of changing patterns.
Kendall (2010)	E1: 16 students E2: 3 music majors	56 audiovisual composite stimuli (7 auditory x 8 visual); 7 audiovisual combinations	Participants sensitive to similar structures presented in the auditory and visual modalities.
Millet et al. (2021)	60 students; 12 per condition	70 s abstract animation from Heider & Simmel (1944); *Adagio* (weak) & *Allegro Marcato* (strong) from Prokofiev's Symphony No.5; Stimuli presented twice.	Presence of music influenced speed of first fixation on film objects & emotion associated with the characters in the abstract animation, but didn't influence attitudes to filmed events.

According to Marshall and Cohen (1988), if music, through ‘structural similarity’, were to have resulted in participants’ attention being directed to a particular feature of the animation while, at the same time, providing particular connotative meaning, then this particular connotation may have become associated with the attended visual feature. Marshall and Cohen (1988, p. 95) also talk of “congruent auditory and visual structure”. However, and importantly, it remains unclear what exactly the authors mean with the phrase “crossmodal (audiovisual) temporal structure”: Are they referring to synchronized elements, correlated temporal, isomorphic inputs, or perhaps something else entirely? Finally, it is worth noting that the animation used in the study had originally been developed by Heider and Simmel (1944) in the context of social psychology research, with participants being asked to describe what was going on by providing some sort of narrative, meaning that this narrative was not generated spontaneously by the participants.

Reflections on the joint process of the emergence of congruent music-visual temporal structure followed by the ascription of meaning (associations) to the visual stimuli resulted in the formulation of the Congruence-Associationist Framework (sometimes referred to as the Congruency-Associationist Model, or CAM for short) for understanding the effects of film music in film and video presentation (albeit with a film showing the spatiotemporal relations between simple elements). Marshall and Cohen (1988) also raise the possibility that this binding of semantic meaning associated with sound onto visual objects presented in the animated film could be considered as a kind of ‘illusory conjunction’ (cf. Treisman & Schmidt, 1982). As a post hoc explanation of the findings obtained, Marshall and Cohen’s suggestions would appear plausible enough (cf. Kahneman & Henik, 1981). That being said, within the CAM framework, the causal relationship between association and congruency remains unclear, that is, whether percepts are associated because they are congruent, or whether they are judged as being congruent because they are associated. Moreover, it is uncertain whether similar occurrences should be considered as a genuine case of multisensory integration or not. Finally, the concept of congruency itself also appears rather ambiguous, unless it is clearly specified in relation to which particular features or dimensions the comparison is being made.

Certainly, the dichotomy between the structure (organization) and (semantic) meaning of the stimuli, no matter whether they happen to be auditory or visual, has some appeal. However, it is unclear whether Marshall and Cohen (1988) ever followed up on their suggestion in order to provide robust support for the claimed mechanism despite the model being re-presented and refined in many subsequent articles. As an aside, one might also note the visual bias that is inherent in Marshall and Cohen’s experimental approach (cf. Hutmacher, 2019) in that one could presumably equally well have asked how visual stimuli affect people’s interpretation of sound, or how audition and vision merge to deliver what Audissino (2017) calls a ‘macro-configuration’. However, that said, the majority of the researchers working in this area to date have tended to focus on people’s interpretation of the visual stimuli.

Cohen (1993) reported a study in which melodies lasting several seconds were sometimes presented at the same time as computerized visual animations showing a single object moving up and down on a screen (looking very much like a bouncing ball). The repeating single melodic notes varied in terms of their tempo (slow, moderate, or fast) and pitch (low, middle, or high), while the tempo (slow, medium, and fast) and height of the bouncing ball (low, medium, and high) were also varied. The participants used a single five-point scale to rate the apparent happiness/sadness of the music and video examples when presented individually. As might have been expected, given the extensive literature on musical emotion (see Hevner, 1936, 1937; Juslin, 2011; Riggs, 1964), the background melody was judged as happier when the tempo was faster and when the pitch was higher. Meanwhile, the rated ‘happiness’ of the bouncing ball increased for faster tempos of bouncing as well as for those bouncing videos where the ‘ball’ bounced higher.

The findings do not appear to support the conclusion subsequently drawn by Cohen (2005, pp. 20–21), namely that: “the meaning of the music and visual materials was systematically related to physical characteristics of the sound and light patterns.” That the small number of participants in a forced-choice task consensually rate physical stimuli along some ‘arbitrary’ response scale, in-and-of-itself tells you absolutely nothing about what those stimuli ‘mean’. At least not if by ‘meaning’, one considers the associations that are spontaneously brought to the top of mind. Certainly, one might worry about the possibility of halo-dumping (Clark & Lawless, 1994); this the name given for the tendency of participants to dump their feelings and experience onto whatever response scale they have been presented with, no matter whether those scales capture their experience or not.

Cohen’s (2005) study also demonstrated a significant crossmodal influence of the music on participants’ rating of the dynamic visual stimuli. So, for example, a ball that bounced high and fast was judged as looking very happy when the accompanying background music happened to be high pitched and fast as well, but was judged as looking less happy when paired with low-pitched and slow background music instead. Taken together, these results therefore appear to show that the happiness attributed to the accompanying music (mediated by variations in tempo and pitch height) influenced the judged happiness of the bouncing ball. What remains unclear, at least from more of a philosophical angle, is whether this should be considered as an example of one audiovisual object/event, or as separate visual and auditory objects/events. However, as a starting point in this debate, one might point to the absence of precisely synchronized or correlated inputs as likely reducing the likelihood of any kind of perceptual unification (see also Spence & Di Stefano, 2025a).

Some researchers have explicitly manipulated the synchrony of the auditory and visual elements in audiovisual film clips (e.g., Iwamiya, 1992, 1994, 2013; see also Bruce Nauman’s (1969) Lip Sync video installation: https://www.moma.org/collection/works/107669). So, for example, Iwamiya (1994) presented short audiovisual film clips that were either synchronized or else had been deliberately desynchronized by 500 ms (note the shift from mid-level, or structural, correspondences to more complex correspondences). The participants had to respond on 22 different bipolar adjective pairs that could be applied to auditory and visual elements of the commercial films selected for this study. It is, however, worth bearing in mind the very large number of semantic differential scales that each participant had to complete in Iwamiya’s Experiment 1 – namely, 40 film clips × 22 SD scales (for four of five conditions) (auditory rating without video; video rating without sound; sound and video ratings in the presence of the other modality) and then rating the degree of matching of sound and video for all clips on a 7-step scale) = 3,560 ratings in total per participant. Factor analysis of participants’ semantic differential responses was interpreted as revealing five dimensions of meaning: Tightness, evaluation, brightness, cleanness, and uniqueness. The results revealed that the participants rated the original synchronized audiovisual clips as matching better than the clips that had been deliberately desynchronized. That said, it should be noted how people rapidly adapt to asynchronous audiovisual speech stimuli, even when the magnitude of the asynchrony is quite large (i.e., several hundred ms; e.g., Dixon & Spitz, 1980; Macaluso et al., 2004; Nahrstedt, 2024; Steinmetz, 1996).

Sirius and Clarke (1994) used a modified version of the approach first presented by Marshall and Cohen (1988), once again using the semantic differential technique to measure people’s ratings of various combinations of auditory and visual stimuli. In this case, computer-generated moving images and music that had been specially composed were used as stimuli. Twenty-seven participants were divided into three groups, with each group exposed to audiovisual (10), visual-only (8), or auditory-only stimuli (9). The results indicated that music exerted a consistent, additive effect on the evaluation of visual stimuli, but no significant interaction effects were documented between specific musical styles and visual sequences. In this case, the absence of any synergistic audiovisual meanings was attributed to the simplicity of the visual material that was presented. Sirius and Clarke proposed an interpretative framework grounded in principles of ecological social perception to account for the observed patterns in crossmodal evaluation. However, with the benefit of hindsight, this study once again appears to be statistically underpowered, especially given the between-participants nature of the experimental design (see Brysbaert, 2019).

Lipscomb and Kim (2004) investigated the relationship between the auditory and visual components of an audiovisual composite. The 28 participants in this study rated the perceived degree of ‘match’ between audio/video components in a series of randomly presented audiovisual composites. (It is, though, unclear whether such a ‘matching’ task can provide meaningful insights into the ‘congruency’ between the stimuli.) The audio parameters that were manipulated included pitch, loudness, timbre, and duration, while the visual parameters that were manipulated included colour, vertical location, shape, and size. Audiovisual composites were created by combining all possible pairs of audio and visual stimuli using three different magnitudes of change (small, moderate, large), giving rise to 48 stimuli in total. The participants’ mean response ratings revealed the following primary relationships: pitch with vertical location, loudness with size, and timbre with shape. Note that the colour was equally matched by the participants with both pitch and loudness.

Kim and Iwamiya (2008) studied people’s perception of simple auditory stimuli and moving letter patterns (referred to as ‘Telops’; that is, animated text on a display as might be seen in television commercials). The studies, which involved participants completing 22 semantic differential ratings scales for each of 64 stimuli (eight auditory patterns crossed with eight Telops patterns), revealed a sensitivity to similar patterns of motion across the visual and audio modalities (see also Kendall, 2010; Lipscomb, 2005). Once again, the semantic differential approach, based on the three dimensions of Evaluation, Potency, and Activity, was used. Two types of formal congruency were found to be effective in creating subjective congruency: Specifically, the synchronization of temporal structures and the matching of changing patterns of auditory and visual events. The former relies on correspondence of the onsets of sound and Telops pattern (i.e., temporal synchrony), the latter, typically, on the combinations of the gradually rising of some acoustic feature, for example, loudness or pitch of sounds, and the expanding (or approaching) of the Telops patterns. The combinations of the gradually rising loudness, or increasing pitch, of sounds and the expanding (or approaching) of the Telops pattern were rated as matching. Formal congruency also contributed to enhancing the evaluation of the audiovisual productions by the participants.

Kendall (2010) conducted a couple of experiments to assess the impact of music in and the perception of visual animation. In a first experiment, seven auditory structures were combined with eight visual patterns (i.e., a total of 56 patterns were presented). The results demonstrated that participants were sensitive to similar structures being presented in the two modalities, such as an arch, ramp, and undulation. However, there appeared to be a preference for left-to-right interpretation of stimuli. In a second study, seven audiovisual contours were presented and participants rated the match between the arch structures in both senses as very good (see also Karwoski, Odbert, & Osgood, 1942).

Finally, Millet, Chattah, and Ahn (2021) conducted a mixed-design experiment in which 60 participants viewed a 70-s dynamic visual animation from Heider and Simmel (1944) while listening to different pieces of music. They chose the same two movements from Prokofiev’s Symphony No. 5, as has first been used in Marshall and Cohen’s (1988) preliminary study. The participants listened to each piece of music by itself; they viewed the animation in silence; and they watched the video when paired with each piece of music. These five within-participant conditions were presented to participants in a randomized order. Participants’ eye position was monitored, as was electrodermal activity, their self-reported emotional state, perception of the film characters, and the three dimensions of the semantic differential scale. Millet et al. assessed the effect of music on participants’ visual attention, their emotional responses, and their attitude towards film objects, and the continuation of narratives. The authors assessed the impact of the latter on participants’ visual attention and their affective responses to the animation. As had originally been suggested by Marshall and Cohen (1988), the presentation of the music while watching the video led to faster first fixations on the visual objects depicted in the animation and supplied emotional content, increasing positive sentiment for the animation’s characters. As such, these results can be taken as providing support for crossmodal spatial attention, driven by structural correspondence between the auditory and visual channels, playing some role in influencing people’s perception of the visual animation (see Ansani et al., 2020, for a similar approach involving a short video made up of static images; and Clemente, Friberg, & Holzapfel, 2023).

### Interim synthesis: Mechanisms underlying reported effects

Taken together, the studies reviewed above point to a number of shared explanatory accounts that extend beyond their original framing in terms of film music or audiovisual matching (e.g., Cohen, 2005), and instead invite interpretation in terms of crossmodal perceptual organization (see Spence & Di Stefano, 2025b). What is noticeable from this review of the literature is how widely Osgood, Suci, and Tannenbaum’s (1957) semantic differential technique has been used by the researchers working in this area (e.g., Iwamiya, 1994; Marshall & Cohen, 1988; Sirius & Clarke, 1994). At the same time, however, it is also worth noting that some film study researchers have criticized this approach, arguing that forcing people to respond along a small (or even large) set of bipolar adjective scales may fail to capture the richness of any emergent properties or sensations that may result from combining the senses (e.g., Audissino, 2017; Wells, 1980; see also Spence & Di Stefano, 2025b).

In addition to the above-mentioned ‘Congruency-Associationist Model’ (Cohen, 2005), and a more conventional cognitive, or experimental, psychology approach (often using the semantic differential technique), various additional explanations for the crossmodal effects have been observed in the reviewed literature. First, any affect (or emotional response) that happens to be associated with, or triggered by, the music may carry over (perhaps as a result of sensory transfer) to influence the viewer’s interpretation of visuals (as in the case of film music, see Spence & Di Stefano, 2025c). Second, there might be some form of crossmodal influence such that visual stimuli appear different as a result of crossmodal perceptual interactions (crossmodal perceptual effects, e.g., Armontrout, Schutz, & Kubovy, 2009; Olivers & Van der Burg, 2008; for a review, see Spence, 2018b). Relevant here, crossmodal research has revealed evidence of extensive crossmodal influences on perceptual grouping (for reviews, see Spence, 2015; Spence & Di Stefano, 2025a; Spence, Sanabria, & Soto-Faraco, 2007), albeit in the absence of any genuine ‘inter-sensory Gestalten’ (see Gilbert, 1938). This can be considered as similar to what Kubovy and Yu (2012, p. 963) term ‘trans-modal gestalts’. In such cases, note, it might be the difference in the perception of the visual stimuli that leads to the change in participants’ ratings. Third, synchronized crossmodal inputs may capture a viewer’s visual attention (see Millet et al., 2021, for some preliminary answers here). While synchronous transients have sometimes been shown to be important, a looser definition of synchrony has been shown to be sufficient to give rise to crossmodal effects at the level of temporally extended stimulus sequences (including correlated accent structures across the senses).

## Crossmodal grouping and perceptual organization

A central question emerging from the reviewed literature on audiovisual integration concerns how and under what conditions signals from different sensory modalities are grouped into a single perceptual event. This section focuses on crossmodal grouping at a perceptual level, with the aim of identifying a number of organizing principles that recur across the empirical literature and constrain how audiovisual integration can occur. Rather than treating synchrony as a sufficient condition for audiovisual binding, it will be clear that temporal alignment between auditory and visual streams does not exert a uniform effect across emotions, but instead interacts with the expressive qualities of the stimuli. This pattern supports a Gestalt interpretation of synchrony as a context-sensitive cue the perceptual consequences of which depend on higher-order structure rather than on simultaneity alone.

### Temporal synchrony as a basic grouping cue

A series of studies on the so-called ‘pip-and-pop’ effect in the field of cognitive psychology have demonstrated that the mere audiovisual synchrony of sound and visual stimuli can lead to the ‘pop-out’ of the latter in complex dynamic visual displays (e.g., Klapetek, Ngo, & Spence, 2012; Van der Burg et al., 2010; Van der Burg et al., 2008). This effect refers to the phenomenon whereby the presentation of a sudden-onset sound (or other transient, such as a tactile stimulus) leads to the pop-out of a synchronized change in a complex visual array of changing items, thus making the synchronized visual stimulus appear more perceptually salient. Such contemporary crossmodal research findings, and many others like them, demonstrate how synchronized audiovisual stimuli can give rise to measurable crossmodal effects on visual perception (see also Grassi & Casco, 2010; Ryan, 1940; Shams, Kamitani, & Shimojo, 2000; Staal & Donderi, 1983).

Moving to real-world examples involving both mid-level and complex stimuli, it is interesting to observe, with Strachan (2006), the synchrony of the elements in Daft Punk’s 1997 house music track *Around the World*. In the video that accompanies the song, the appearance of four sets of four dancers are synchronized to coincide with (and thus represent) different parts of the music. According to Strachan (2006, p. 200), each musical unit “is choreographed to a particular group of dancers and dance moves in the video in a kinetic representation of musical structure, melody, and harmony.” Indeed, music videos may more frequently capture the temporal aspects of the music by synchronized visuals than other forms of audiovisual media.

While these examples help to illustrate how synchrony is one of the most effective ways to induce a sense of objective unity in the observer or to establish a causal link between multiple sensory inputs, the effectiveness of synchrony is strongest for discrete, punctate events having a clearly defined onset. Under these conditions, synchrony can operate as a powerful bottom-up cue that captures attention and facilitates perceptual binding. At the same time, this strength also highlights a limitation: temporal synchrony alone does not scale well to extended or structurally complex stimuli, such as sequences, rhythms, or continuous audiovisual streams. In these cases, simultaneity at isolated time points is insufficient to account for stable perceptual grouping.

### Beyond simultaneity: Correlated temporal structure and isomorphism

In many mid-level audiovisual displays, especially those involving extended sequences, binding does not depend on millisecond-level simultaneity. Instead, correlated temporal structures (e.g., shared rhythm, accent patterns, or dynamic contours) are sufficient to support perceptual grouping (see Iwamiya, Sugano, & Kouda, 2000; Lipscomb, 2013; Müller, 2010). From a Gestalt perspective, the same temporal form or rhythm can be instantiated in different sensory modalities, giving rise to what has often been described as structural or isomorphic correspondence. As observed by Pratt:

“An auditory rhythm is auditory, and that's that; but the same rhythm – a Gestalt – may also be visual or tactual, and the graceful lilt, let us say of a waltz rhythm […] will be present in all three modalities. Gestalten […] reveal innumerable iconic relations and resemblances across modalities. Therein lies the great power of art, for the moods and feelings of mankind are capable of iconic *presentation* in visual and auditory patterns a mode obviously far more direct and effective than symbolic *representation*.” (Pratt, 1969, pp. 25–26 [italics in original]; see also Collopy, 2000; Kulezic-Wilson, 2004).

Recently, Di Stefano and Spence (2025) reviewed the literature supporting the existence of similar temporal grouping principles in both audition and vision (e.g., Aksentijević, Elliott, & Barber, 2001; Allen, Walker, Symonds, & Marcell, 1977; Kang, Lancelin, & Pressnitzer, 2018; Marks, 1987). This body of empirical research demonstrates that temporal patterns that are perceived in one sensory modality – such as audition – can often be recognized when presented via another sensory modality, such as vision. While this crossmodal recognition is most robust between audition and vision, it may sometimes extend to the sense of touch, though it is far less evident in the chemical senses. However, when considering the perception of temporal structure distributed across modalities (one form of intersensory Gestalten) – where some portions of the temporal information are presented to one sensory modality and the remainder to another – the evidence becomes sparse and less conclusive (though see Huang et al., 2012; for a review, see Spence, 2015).

At one level more of abstraction, the relations between multi-element (or dynamically changing) auditory and visual stimuli may be isomorphic (cf. Omwake, 1940; Ravignani & Sonnweber, 2017; Thornley Head, 2006). Here, for example, we might perhaps think of transposing a temporal auditory pattern into a spatial visual arrangement (though what constitutes the most appropriate transformation between the auditory and visual modalities undoubtedly remains something of a contentious issue: see Handel, 1988a, b; Julesz & Hirsh, 1972; Kubovy, 1988). Crucially, isomorphism involves a formal structural correspondence between elements, and thus requires a clearly defined criterion or mapping rule – something that makes it different from mere perceptual similarity based on the sharing of phenomenological properties (see also Schöffer, 1985; Vanel, 2009). As Di Stefano and Spence (2024) note, while judgements of perceptual similarity are relatively easy to understand within a single sensory modality, they become considerably more difficult to define and apply crossmodally. This is likely because different senses often rely on distinct representational codes and processing mechanisms, and lack a common metric, thus making direct comparisons ambiguous and highly dependent on context, task demands, and perhaps also individual experience. One might question the relation between isomorphism and metaphor (Dahl & Adachi, 2013; Liu & Kennedy, 1997; Ramachandran et al., 2020; Wagner, Winner, Cicchetti, & Gardner, 1981). However, it should be noted that getting to grips with this thorny question lies beyond the scope of the present article.

In an intriguing early article, Julesz and Hirsh (1972) looked for analogies between auditory and visual pattern perception (see also Harris, 1950; Stevens, 1958). They consider the various Gestalt grouping principles and consider the extent to which they may be present in both the visual and auditory modalities. They also suggested that grouping by proximity and similarity likely operate in both senses, and that good continuation (or smooth transformation) also seems to apply to both senses, as does figure/ground segregation. Numerosity and matching spatiotemporal patterns (perhaps involving melody) could be present in both senses. Set effects too can presumably be extended across the senses (Liu, 1976). By contrast, symmetry and closure appear more relevant to the visual rather than the auditory modality, and emergence has been documented in the visual modality but is not so obviously apparent in audition.

There is also an intriguing literature emerging on morphodynamics, connecting time-varying sounds/music with complex visual forms. For instance, Wanke et al. (2025) recently investigated the audiovisual associations between contemporary experimental music and abstract shapes. The authors chose sonic compositions belonging to specific styles of experimental music, namely spectralism and electronic-glitch music. These compositions are typically perceived as a sequence of sound patterns which can prompt a phenomenological and sensory engagement (Wanke, 2021) rooted in processing mechanisms associated with the early stages of perception, such as primal scene segregation (Bregman, 1990) and the formation of auditory Gestalten (Koelsch & Siebel, 2005). Overall, Wanke et al.’s results revealed that participants consistently selected images that shared a large number of morphological features with the corresponding auditory stimuli. This not only reveals a general preference for linear spectrotemporal visual representations of auditory experiences (Wanke, 2023), but also supports the notion that listeners spontaneously transpose auditory patterns into spatial visual forms.

### Emergence of multisensory perceptual unit in the context of structured audiovisual stimuli

In some cases, the combination of structured audiovisual stimuli has been interpreted in terms of a multisensory perceptual unit (e.g., Kim & Iwamiya, 2008). This outcome is believed to occur especially in the case of functionally congruent stimuli, such as music and images in movies, where neither perfect synchrony nor any alternative structural congruence seem to apply. Beyond temporal congruency (Bolivar, Cohen, & Fentress, 1994), emotional congruency has also been evoked as an effective means to induce the emergence of a multisensory unit. This occurs when two sensory inputs, taken separately, are associated with the same or similar emotional meaning. Note that, for the binding of the two sensory inputs to occur, they have to be temporally aligned and/or the inputs available to each sensory modality should be temporally correlated with one another (Parise et al., 2012, 2013; see also Jertberg et al., 2024).

Importantly, the kind of multisensory emergence just discussed cannot be explained solely by temporal synchrony (§3.1) or structural isomorphism (§3.2). Although synchrony may play a facilitatory role, what distinguishes emergent audiovisual experiences is the superadditive unity and dynamic interplay of the various elements. That is, the audiovisual whole expresses something that neither sensory modality conveys by itself, and which is seemingly more than the mere sum of the parts (i.e., of the two modalities when considered individually; though see Spence, 2025). This may involve affective or conceptual layering, narrative construction, or spatial-temporal abstraction, often guided more by expressive fit than by precise temporal alignment.

### Multiple outcomes of audiovisual co-presentation

**Fig. 2:**
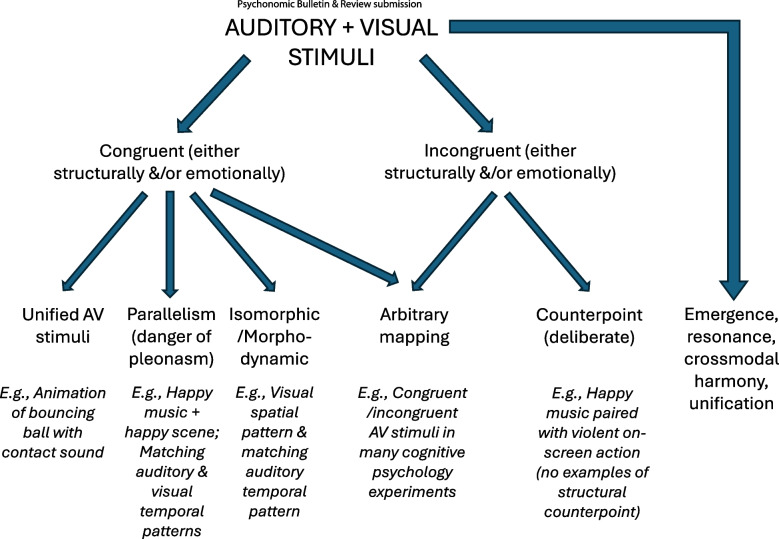
Possible outcomes when mid-level auditory and visual stimuli are presented

Figure 2 highlights some of the various outcomes that may result when independent auditory and visual stimulus streams are presented together. Taken together, the literature reviewed here suggests that audiovisual co-presentation can lead to several distinct perceptual outcomes, including perceptual binding, in which signals are grouped into a single multisensory event; attentional modulation, where one modality enhances processing in another without full integration; partial interaction, such as biasing or priming effects across modalities; perceptual independence, where signals remain segregated despite temporal alignment; competition or interference, particularly when crossmodal cues conflict. This underscores the fact that occurrences of temporal synchrony and structural isomorphisms in the context of mid-level audiovisual correspondences are best understood as constraints on possible outcomes, rather than as mechanisms that uniquely determine integration.

Notice how a number of these possible outcomes have been reviewed in depth elsewhere (e.g., see Spence & Di Stefano, 2025a, b, c). Synchrony is clearly an important cue to binding (working best with transients occurring simultaneously in both modalities). However, it is important to recognize how beat/accent structure also provides grounds for the synchronization of sequences of unimodal sensory signals (e.g., Kendall, 2010; Lipscomb, 2013). There is, however, also a separate more recent experimental literature on correlated inputs (Parise et al., 2012, 2013) that provides an even more powerful cue to crossmodal binding. Subliminal audiovisual temporal congruency in music videos enhances perceptual pleasure (Lin, Yeh, & Shams, 2022). One might also wonder with regard to the context of emotive music, whether precise sensory synchronization matters, or whether instead it is the simultaneity of the emotions evoked by the music that are key to biasing the interpretation of any visual stimuli (cf. Su, 2014).

## Discussion

The research that has been reviewed in this narrative historical review primarily concerns those crossmodal interactions involving mid-level audiovisual crossmodal correspondences (see Spence & Di Stefano, 2025b), as well as a few cross-level examples involving mid-level stimuli in one modality and complex stimuli in the other. While some of the early findings in this area were taken to inform film music studies (e.g., Cohen, 2005), our view is that they may actually be more informative with regard to (and/or better explained in terms of) crossmodal Gestalt perceptual grouping (see also Daurer, 2010; Staal & Donderi, 1983; Welch, DuttonHurt, & Warren, 1986; for review, see Spence & Di Stefano, 2025a). Certainly, stimulus structure (namely synchronized audiovisual inputs and correlated auditory and visual signals) appears to play an important role in the case of crossmodal perceptual grouping (Parise et al., 2012, 2013; see also Müller, 2010).

**Fig. 3:**
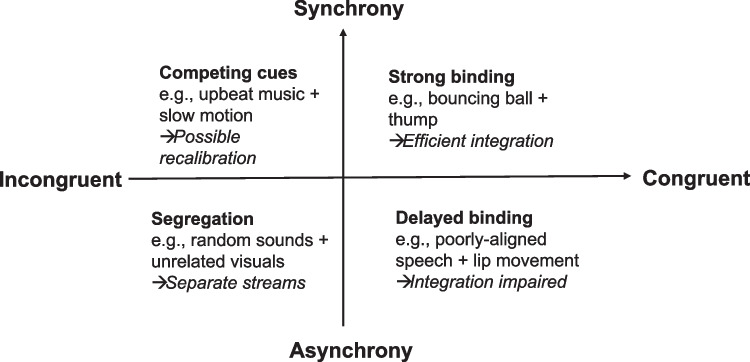
Possible outcomes when mid-level auditory and visual stimuli (varying in terms of their synchrony and congruency) are presented

The literature on mid-level (or structural) crossmodal correspondences shares much with the cognitive psychology literature investigating the effects of pairing music with paintings (for a review, see Spence, 2020), or else with short movie clips (Spence & Di Stefano, 2025c), in that those participants taking part in these studies are rarely, if ever, told anything about why exactly they are being presented with the particular combinations of auditory and visual stimuli that the experimenter(s) has/have settled on. Given that there is no obvious intentionality behind combining mismatching auditory and visual stimuli, any possibility of meaningful counterpoint is presumably lost (see Spence & Di Stefano, 2025b). However, one difference that comes out in this particular literature is the importance of the various different kinds of temporal alignment that may be experienced (see Fig. 3). From this perspective, temporal alignment should not be treated as a unitary construct, but rather as a family of relations that constrain, without determining, the outcome of audiovisual co-presentation.

Vision typically dominates over audition in laboratory-based experimental psychology research. This is thought to be due to the ‘greater cortical real estate’, information processing bandwidth, and attention that are apparently available to the visual modality (Gallace et al., 2012; Heilig, 1992; Posner, Nissen, & Klein, 1976). The dominant approach in this area involves assessing how music affects visual perception, rather than vice versa. This asymmetry reflects both theoretical assumptions and practical methodological choices, rather than a principled claim about multisensory perception. Such an asymmetry is, however, consistent with the focus on vision that has been highlighted in multisensory research more generally (Hutmacher, 2019). While the visual stimuli in the research reviewed here have mostly been mid-level (except Iwamiya, 1994), i.e., involving multiple, possibly dynamically changing simple stimuli, the auditory stimuli have included both mid-level and more complex semantically/emotionally meaningful music clips. It may be for this reason that auditory stimuli have typically been found to dominate visual stimuli in this literature (see Vines et al., 2011, for evidence that semantically meaningful visuals can sometimes impact people’s auditory perception too). As Embler (1974) noted more than half a century ago: “music and film each depend upon the phenomena of movement and are thereby allied aesthetically…sound movement reinforces visual movement.”

One other important methodological point to consider here relates to the widespread use of the semantic differential technique (e.g., Iwamiya, 1994; Iwamiya et al., 2000; Marshall & Cohen, 1988; Millet et al., 2021; Sirius & Clarke, 1994), though, as has been mentioned already, some commentators have criticized how this popular experimental approach, imported from cognitive psychology, may ultimately fail to capture important aspects of meaning, such as any emergent macro-structural or superadditive responses (Audissino, 2017). As such, there may be occasions in which a more open (and hence less constrained) response format is more appropriate for those wanting to know how people’s responses to dynamic visual displays (such as animations) may be changed by the simultaneous presentation of sound (as can be found in a subset of the studies of mood music in the context of film studies; for a review, see Spence & Di Stefano, 2025b).

A related methodological issue concerns the frequent conflation of stimulus co-presentation with stimulus coherence. In many experimental designs, auditory and visual stimuli are presented simultaneously (or within a limited temporal window) precisely because the goal is to assess crossmodal effects. However, such designs make it difficult to disentangle the effects of mere co-presentation from those arising from genuine structural, temporal, or expressive relations between the component stimuli. As a result, observed crossmodal influences may reflect the presence of concurrent multisensory input rather than the specific coherence or congruency of the audiovisual pairings.

Taken together, these considerations highlight the need for a more nuanced account of temporal alignment and congruency in audiovisual perception in order to accommodate both low-level synchrony and higher-order structural and expressive relations.

### Future directions

While the research that has been published to date highlights the importance of synchrony in establishing mid-level crossmodal correspondences, future research should clarify the specific contribution of precise temporal alignment relative to other factors, such as structural isomorphism, temporally correlated inputs, and semantic relatedness, in the perception of audiovisual correspondences. Moreover, it is crucial to examine how specific musical features (e.g., tempo, pitch, rhythm) interact with dynamic visual elements (e.g., motion, speed, direction) to influence people’s perception and any emotional response that might be evoked by the stimuli that happen to be presented.

An important direction for future research concerns the systematic disentangling of stimulus co-presentation from crossmodal coherence. While many existing studies have chosen to focus on the simultaneous or temporally proximate presentation of auditory and visual stimuli in order to elicit crossmodal effects, future experimental designs could benefit from independently manipulating the presence of concurrent multisensory input and the degree of structural, temporal, or expressive correspondence between modalities. Such approaches would make it possible to assess not only whether audiovisual co-presentation influences perception, but under which conditions coherence constrains perceptual grouping and interpretation.

In the era of the replication crisis in psychological and other sciences, the very small sample sizes used in many of the early between-participants studies relevant to the study of mid-level crossmodal correspondences would not be accepted today. Thus, another important direction looks back to published literature with the aim of replicating some of the foundational observations in this particular area of research with adequate sample sizes (e.g., Marshall & Cohen, 1988; Sirius & Clarke, 1994).

In parallel, there is a need to determine the cognitive mechanism(s) by which music influences people’s perception of, and responses to, dynamic visual content, likely involving some combination of subjective report and psychophysiological measures (cf. Thaiwong & Fukumoto, 2024, 2025). Subjective reports, including continuous ratings and semantic differential scales, can capture the nuances of conscious experience, while techniques like eye-tracking, electrodermal activity, and heart-rate variability can provide insights into attentional and emotional responses. Furthermore, neuroimaging techniques could potentially be used to investigate the neural mechanisms underlying the integration of synchronous versus asynchronous, and congruent versus incongruent, audiovisual information at different levels of processing (e.g., expecting higher gamma band oscillations in response to congruent/synchronous audiovisual stimuli, based on the available evidence on separate senses: see Knief et al., 2000; Müller et al., 1997). Understanding how the brain differentiates and integrates these dimensions will be crucial for those wishing to develop any kind of comprehensive model of crossmodal perception involving the more complex configurations of stimuli that we tend to be presented with in everyday life.

Finally, future research should aim to extend the current findings to more ecologically valid scenarios. While controlled laboratory studies have undoubtedly provided researchers with essential insights, examining how synchrony, temporal correlation, and congruency operate in complex real-world contexts, such as film viewing, human-computer interaction, and artistic performances (see Daniels et al., 2010, for many such examples), will be crucial for understanding their broader significance. That said, the latter examples would appear to fall more in the realm of complex rather than mid-level correspondences. This investigation can benefit from the use of portable and low-invasive devices for monitoring physiological parameters, such as cardiac or electrodermal activity.

## Conclusions

This review has examined empirical research on mid-level audiovisual crossmodal correspondences, identifying a set of recurring patterns across otherwise heterogeneous paradigms. Crossmodal effects are most consistently observed when auditory and visual streams exhibit some form of temporal alignment or structural correspondence, whereas simple co-occurrence or semantic association alone often proves insufficient to account for the reported outcomes. Rather than interpreting these findings primarily in terms of film music or modality-specific meanings, we have argued that many such effects are more parsimoniously understood within a framework of crossmodal perceptual organization. From this perspective, temporal alignment, structural similarity, and expressive coherence act as partially independent constraints on perceptual grouping, shaping how multisensory information is integrated over time rather than functioning as independent mechanisms.

By synthesizing results across diverse experimental approaches, this review highlights the importance of distinguishing between the effects of stimulus co-presentation and those that emerge from crossmodal coherence. Note here how making this distinction explicit has implications both for the interpretation of existing findings and for the design of future studies aimed at isolating the conditions under which mid-level audiovisual correspondences emerge. More broadly, an organizational view of audiovisual interaction may help unify research across domains while supporting experimental paradigms that better capture the dynamic and emergent nature of multisensory perception.

## Data Availability

Not applicable.

## Footnotes

1 Synaesthetes are those rare individuals who experience a consistent vivid and automatic concurrent percept in the same or different sensory modality in response to a specific inducer (Deroy & Spence, 2013). For instance, colour-music is a form of synaesthesia in which people see idiosyncratically-matched colours (that are not physically present) in response to specific musical notes and/or timbres.
